# Exploring the over-time, multifaceted impacts of three COVID-19 lockdowns on aspects of capability, wellbeing and mental health across vulnerabilities in Austria

**DOI:** 10.1038/s41598-022-20977-z

**Published:** 2022-10-01

**Authors:** Timea M. Helter, Agata Łaszewska, Judit Simon

**Affiliations:** 1 Department of Health Economics, Center for Public Health, Medical University of Vienna, Kinderspitalgasse 15, 1090 Vienna, Austria; 2 Department of Psychiatry, University of Oxford, Warneford Hospital, Oxford, OX3 7JX UK

**Keywords:** Psychology, Health care, Risk factors

## Abstract

The Austrian government imposed multiple major lockdowns during the COVID-19 pandemic, but the relevant measures and their perceptions varied over time. The aim of this study was to compare the over-time impacts of the three COVID-19 lockdowns between March 2020 and December 2021 for (capability) wellbeing and mental health in Austria. Adult Austrian residents (n = 87) completed an online survey about their experiences during three COVID-19 lockdowns, including capabilities (OxCAP-MH), depression and anxiety (HADS), and general wellbeing (WHO-5). Differences across the baseline and follow-up scores of these instruments were summarised by demographic/socioeconomic characteristics. Longitudinal comparisons of the impacts of the lockdowns were conducted using random effect models on panel data for overall instrument scores and individual capability items. The levels of (capability) wellbeing and mental health decreased for most respondents across the three lockdowns: average 2.4% reduction in OxCAP-MH scores, 18.8% and 9% increases in HADS depression and anxiety subscale scores respectively, and 19.7% reduction in WHO-5 score between the first and third lockdowns. Mental health treatment prior to the pandemic, social support and satisfaction with government measures were the most influential characteristics that determine the association with impacts of the chain of lockdowns. Our study is the first to assess the differential capability limiting aspects of lockdowns over time alongside their impacts on mental health and general wellbeing and calls for special attention for mental health patients, isolation and satisfaction with government measures.

## Introduction

Several waves of lockdowns have been adapted by governments worldwide to limit the spread of the severe acute respiratory syndrome coronavirus 2 (SARS-CoV-2), which causes the COVID-19 disease, since it was declared as a pandemic by the World Health Organisation (WHO) on 11th March 2020^1^. Although the restrictions were adopted for the protection of physical health, the subsequent implications on the mental wellbeing of individuals were beyond expectations^2^. Even the United Nations reported that the COVID-19 pandemic is not only compromising physical health, but is also increasing psychological suffering^3^. The mental health impacts of pandemics can take many different forms, and previous studies reported adverse effects on the mental health of people who were quarantined in the SARS and MERS outbreaks^4^. Similarly, the COVID-19 pandemic has had a major impact on mental health^5,6^. Living in isolation and quarantine had multiple negative effects on the physical and mental health of individuals, including the exacerbation of feelings of anxiety and uncertainty^7^. Empirical findings suggest that at least one out of every five people experiencing medical condition-related isolation reported clinically significant psychological distress, independently of the length of isolation and culture^8^. The most common psychological distress reactions include anxiety, insomnia, perception of insecurity, anger, fear of illness, and risky behaviours^9^. Empirical research related to COVID-19, SARS and MERS identified some key pandemic stressors that can increase the risk of a negative mental health outcome, including exposure not only to the virus, but also to media, death, movement restriction, economic hardship, stigma, prejudice and discrimination, intimate partner violence and child abuse and neglect, and occupations that increase risk of exposure/infection, stress and other challenges to health and wellbeing^10^. The impacts of pandemics therefore go beyond a narrow definition of mental health and affect broader aspects of wellbeing, including the capabilities of what people are free to do or be^11^.

The impacts of the COVID-19 pandemic on mental health are different across people with different socioeconomic characteristics, including an unequal impact of disrupted and delayed access to mental health services^12,13^. People with higher socioeconomic backgrounds were better able to derive some positive perceptions and psychological outcomes from the lockdown period^14^. Lockdowns have also been shown not to have uniformly detrimental effects on mental health and wellbeing because some people are psychologically more resilient to their effects^15^. Our study focusing on the first lockdown period found some significant negative impacts in terms of anxiety, depression, capabilities and general wellbeing linked to identifiable vulnerabilities^11^. Particularly significant deteriorations in capability wellbeing were observed in case of respondents who had mental health treatment prior to the pandemic, directly experienced the COVID-19 disease, or belonged to an ‘at risk’ group for severe COVID-19 symptoms. The pandemic was responsible for the deterioration of many psychiatric disorders including depression, anxiety, OCD, and PTSD^16^. The crisis also added to the stress on healthcare workers and resulted in a reported increase in suicide among physicians across the globe^17^. Due to the multifaceted aspects of the pandemics’ impacts, it is of particular importance to explore the consequences of lockdowns across different vulnerable groups of people. Beyond socioeconomic differences, this includes, for instance, the aspects of unequal occupational hazards, unequal impacts of disrupted access to healthcare services, and unequal impacts of pandemic countermeasures and from regressive policy measures^13^.

While most European states implemented some form of confinement measures to hinder the spread of the disease, the extent of limitations of people’s freedoms differed across individual countries^18^. Austria introduced rather aggressive public health measures in several waves of lockdowns in 2020 and 2021^19^. Among others, this included limitation of social contacts through a curfew, closure of schools and non-essential shops and services, avoidance of case importation, hygiene measures, testing and case tracking^20^. When comparing the compliance with the public health measures across lockdowns, it was found to be generally lower during the second than the first lockdown, but it varied according to the type of the measure^21^. This implies that people had a mixed perception of the public health measures.

The extended consequences of COVID-19 related lockdowns are widespread and pose a potential long-term burden to each society. Some studies estimated that nearly a quarter of the population might suffer from post-traumatic stress disorder following a pandemic^22^. The current pandemic is expected to have worldwide negative impacts on economic and other social determinants of health in the long term, which increase the likelihood of mental health conditions most susceptible to negative social determinants, including anxiety, mood, disorders related to trauma and stress, and even suicide^12^. Evidence shows that after lockdown restrictions being eased, mental health problems do not decrease to a level observed before the pandemic^23^. Social isolation is known to be a risk factor for the manifestation of psychological illnesses^24^. Moreover, there is a lack of longitudinal studies investigating the consequences of lockdowns from a rather holistic perspective, for instance, the capability framework. This approach places emphasis on promoting wellbeing through enabling people to realise their capabilities and engage in behaviours that they value^25^. The capability approach was developed by Amartya Sen with a core focus on what individuals are free and able to do and be (i.e., capable of)^26,27^.

The currently available evidence in terms of mental health and wellbeing impacts beyond the actual lockdowns is contradicting. People affected by the experience of loneliness during the lockdown were shown to be significantly less protected from the effect of lockdown-related stress on subsequent post-lockdown depression^28^. Moreover, subsequent lockdowns pose further mental health burden, especially through isolation and unhealthy life style choices^29^. However, some studies found that the psychological impact of COVID-19 lockdowns was small in magnitude and highly heterogeneous^15^. This suggests that subsequent lockdowns have multifaceted impacts that have not been investigated in the context of different capabilities yet. The aim of this study was to compare the impacts of three COVID-19 lockdowns in Austria in 2020 and 2021 on different aspects of (capability) wellbeing, as well as on mental health.

## Methods

### Data collection and participants

Longitudinal data were collected at three timepoints via the SoSci online survey platform (Version 2)^30^. Participants had to give informed consent at each wave of the data collection. The first wave of data collection was in May/June 2020, with all questions referring to the initial one-month lockdown period in Austria between 15 March and 15 April 2020 (1st wave). Respondents were recruited via convenience sampling during the first wave of data collection by people responding to our survey advert. The advert was distributed via multiple channels including social media platforms (including Facebook, Twitter, WhatsApp) and emails targeting a wide range of individuals and organisations (universities, non-profit organisation such as Red Cross, and local governments) throughout Austria^11^. In order to be eligible to participate in the study, respondents had to declare that they were older than 18 years, had sufficient German knowledge, and were residents in Austria at the time of the COVID-19 outbreak. More information about the first wave of the study can be found in Simon et al.^11^. Respondents were asked to provide their emails addresses if they were willing to participate in subsequent data collections. Those who agreed to the follow-up were contacted again in December 2020 and received the link to the second wave of the survey. The second wave of data collection took place in early December with questions referring to the lockdown measures between 17 November and 6 December 2020 (2nd wave). More information about the second wave of the study can be found in Laszewska et al.^21^. Respondents to the second wave of the data collection were contacted for a third time in December 2021 with questions referring to the lockdown measures between 22 November and 12 December 2021 (3rd wave). Respondents who provided sociodemographic and COVID-19-related information, completed all standardised outcome instruments, and could be matched across the three timepoints, were included in this study.

### Survey

During each timepoint, the online survey consisted of the relevant participant information and consent forms, sociodemographic information, perceptions about the COVID-19 pandemic and relevant lockdown measures. Questions were the same across the three survey waves except for questions related to the lockdown measures which were adapted at each wave of the survey. The final part of the questionnaire consisted of four self-reported standardised and validated outcome instruments.

### Instruments

Capability wellbeing was assessed by the OxCAP-MH, which was originally designed to capture different wellbeing dimensions within the capability framework in the context of mental health outcome measurement across 16 items^30,31^. It covers items of individual well-being including: limitation in daily activities; Social networks; Losing sleep over worry; Enjoying social and recreational activities; Having suitable accommodation; Feeling safe; Likelihood of assault; Likelihood of discrimination; Influencing local decisions; Freedom of expression; Appreciating nature; Respecting and valuing people; Enjoying friendship and support; Self-determination; Imagination and creativity; and Access to interesting activities or employment. Higher OxCAP-MH scores indicate higher capabilities. The OxCAP-MH is scored on a 0–100 scale, with higher scores indicating better capabilities. The OxCAP-MH has shown good psychometric properties including internal consistency (Cronbach’s alpha between 0.79 and 0.85), test–retest reliability (intra class correlation coefficient 0.80), construct validity and responsiveness in both English and German populations^32,33^.

Mental health was evaluated by the HADS instrument that is divided into Anxiety (HADS-A) and Depression (HADS-D) subscales both containing seven items scored on a four-point scale from zero (not present) to three (considerable)^34^. Both the HADS-A and HADS-D subscales are scored from 0 to 21, with higher scores indicating higher anxiety or depression levels. The HADS has good psychometric properties in assessing the presence and severity of anxiety disorders and depression in both somatic and psychiatric cases, also beyond the hospital setting, including the primary care patients and general population. Its Cronbach’s alpha coefficients of internal consistency are between 0.68 and 0.93 for HADS-A and between 0.67 and 0.90 for HADS-D^35^. General wellbeing was assessed by the WHO-5, which has five items including feeling cheerful and in good spirits, feeling calm and relaxed, feeling active and vigorous, waking up feeling fresh and rested and daily life being filled with interesting things^36,37^. The WHO-5 is scored 0–25, with higher scores representing higher well-being. A review of 213 studies using the WHO-5 as an outcome measure verified the instrument's construct validity, responsiveness, and suitability as a screening instrument for depression^37^. The Cronbach’s alpha for mental health patients is 0.83 in case of depression^38^. Social support, assessed by the MSPSS questionnaire, was used as one of the resilience group indicators based on the findings of the first wave of the study^11^. The MSPSS is a self-reported measure of subjectively assessed social support with good internal consistency (Cronbach’s alpha 0.88)^39^. Respondents were divided into two groups, including low- to moderate and high social support, defined as ≤ 5 and > 5 on the 1–7 point scale, respectively^39^.

### Statistical analysis

(Capability) wellbeing and mental health were compared across socioeconomic and other characteristics^11,21^. These included levels of social support, mental health treatment prior to the pandemic, belonging to an ‘at risk’ group (in terms of vulnerabilities, e.g. aged above 65 years or selected physical health conditions), being defined as key worker, knowing someone who died of COVID-19, and perception of government interventions (positive/negative). Anonymous data were extracted from the three waves of the online survey and checked for logical inconsistencies (e.g. time taken to complete the survey) and completeness prior to inclusion in the analysis.

Instrument scores at each timepoint were summarised by demographic and socioeconomics characteristics. Wilcoxon signed-rank test or ANOVA were conducted to assess whether mean instrument score ranks differed across the subgroups at each timepoint. Next, a set of analyses employed random effect models on panel data to examine how demographic and socioeconomics characteristics explained change in the instrument scores across the three waves of data collection. The OxCAP-MH, HADS-D, HADS-A and WHO-5 change scores were used as dependent variables and the sociodemographic characteristics as independent variables. Changes captured by individual capability wellbeing items were assessed across the three lockdowns through changes in nominal and ranking scores and alongside their associations with demographic and socioeconomic characteristics. Associations related to the OxCAP-MH items were investigated in more details to explore the impact of lockdowns on different capability dimensions, including item level scores and their rankings. The panel data analysis using random effect models was repeated for the items of the OxCAP-MH instrument as dependent variables. Significance level of p < 0.05 was considered in all analyses. Analyses were conducted on complete cases (i.e. item scores available for each standardised instrument) in STATA v.16^40^.

### Ethical approval and consent to participate

The study and experimental protocols were approved by the Ethics Committee of the Medical University of Vienna on 26 May 2020 (EK Nr: 1529/2020). Informed consent was obtained from all individual participants included in the study. All procedures performed in studies involving human participants were in accordance with the ethical standards of the Ethics Commission of the Medical University of Vienna (EK 1529/2020) and with the 1964 Helsinki declaration and its later amendments or comparable ethical standards.

## Results

**Figure 1:**
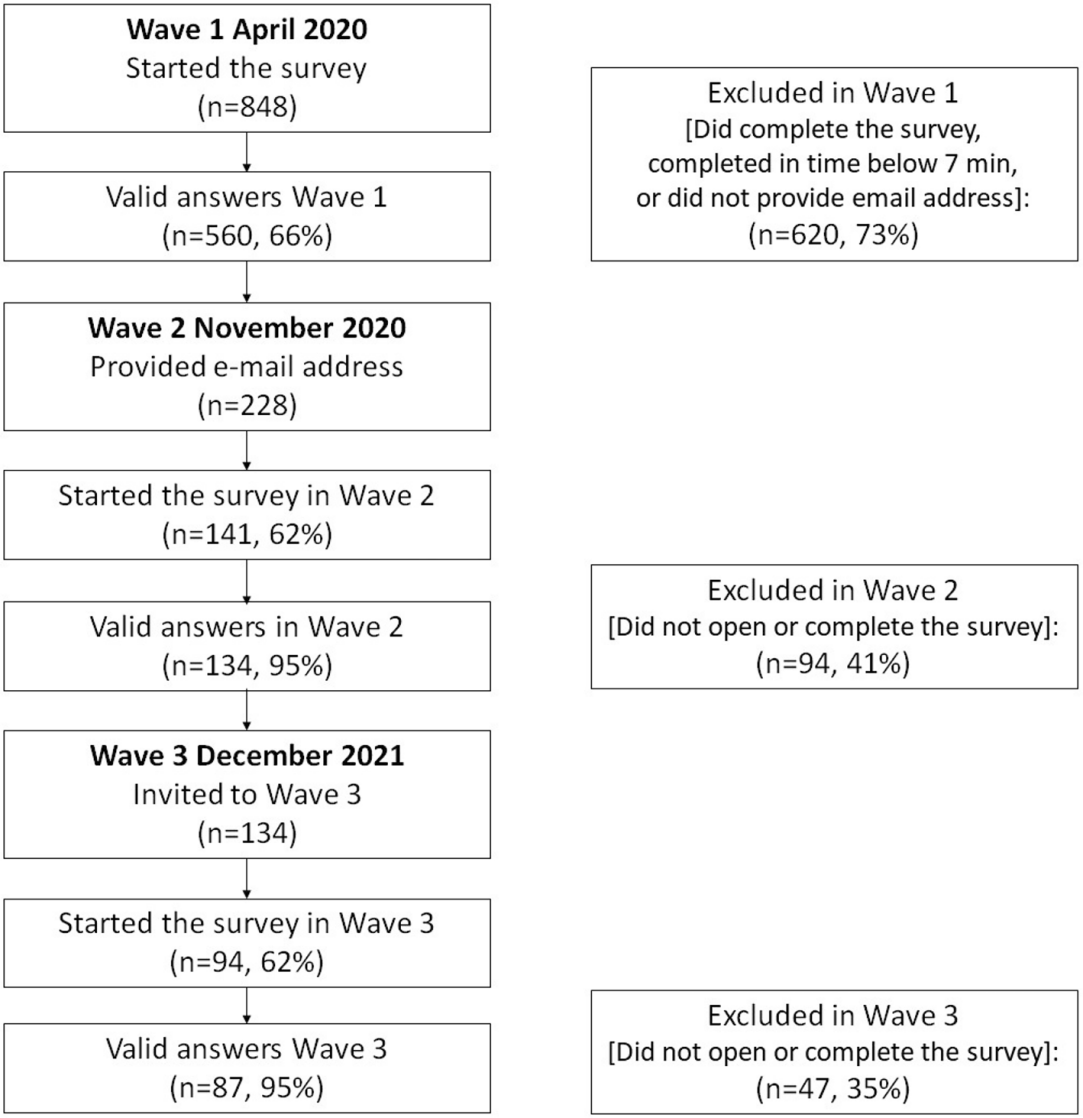
Recruitment of participants.

The first wave of the study had 560 participants, 228 of those provided their email addresses and were contacted later. The second wave of the survey was completed by 134 persons who were contacted for participation in the third wave. Out of the 94 participants who started the survey, 87 provided valid answers and were included in the current analyses (75% female, mean age *M* = 45.08 years, *SD* = 12.74). The recruitment of participants is shown on Fig. 1.

The majority of participants were employed (n = 62; 71%). Further characteristics of the survey cohort are listed in Table 1 alongside the different instrument scores by sociodemographic.

**Table 1:** Baseline, follow-up and change scores of capability wellbeing, mental health, and general wellbeing outcomes across the three lockdowns by demographic and socioeconomic characteristics.

	n	%	OxCAP-MH			HADS-D			HADS-A			WHO-5		
			1st wave	2nd wave	3rd wave	1st wave	2nd wave	3rd wave	1st wave	2nd wave	3rd wave	1st wave	2nd wave	3rd wave
**Full cohort**	87	100	73.72 (12.09)	73.71 (12.94)	71.95 (13.28)	5.05 (4.27)	5.52 (4.38)	6.00 (3.96)	6.66 (4.26)	6.56 (4.26)	7.26 (4.56)	15.16 (5.07)	12.87 (6.10)	12.18 (5.99)
**Gender**														
Female	65	75	74.38 (12.01)	73.56 (12.89)	71.88 (12.77)	4.95 (4.08)	5.48 (4.36)	6.05 (3.85)	6.62 (4.10)	6.60 (4.15)	7.65 (4.41)	15.11 (5.03)	12.60 (6.11)	**11.35 (5.73)**
Male	22	25	71.80 (12.38)	74.15 (13.41)	72.16 (14.99)	5.32 (4.87)	5.64 (4.55)	5.86 (4.37)	6.77 (4.81)	6.45 (4.66)	6.14 (4.93)	15.32 (5.28)	13.68 (6.13)	**14.64 (6.22)**
**Age**														
18–29	12	14	77.60 (10.89)	74.48 (14.19)	74.74 (72.86)	5.42 (4.54)	6.25 (4.14)	5.33 (2.87)	7.75 (4.41)	7.92 (4.38)	8.33 (5.18)	15.00 (5.08)	12.00 (11.00)	12.00 (12.50)
30–49	35	40	72.45 (13.01)	73.21 (10.71)	72.86 (13.35)	5.40 (4.62)	5.86 (4.54)	6.17 (4.44)	7.20 (4.99)	6.83 (4.71)	7.23 (4.89)	14.51 (5.37)	12.43 (5.89)	11.97 (6.09)
50–64	38	44	73.23 (11.74)	73.40 (14.77)	69.61 (14.22)	4.79 (3.95)	5.16 (4.41)	6.18 (3.90)	6.00 (3.42)	6.08 (3.79)	7.18 (4.08)	15.58 (4.89)	13.53 (6.31)	11.95 (5.99)
65–79	2	2	82.03 (3.31)	83.59 (3.31)	83.59 (1.10)	1.50 (0.71)	2.00 (1.41)	3.50 (2.12)	3.00 (1.41)	3.00 (0.00)	3.00 (4.24)	19.50 (0.71)	19.50 (0.71)	18.50 (0.71)
**Migration background**														
Yes	10	11	70.00 (13.46)	77.34 (12.78)	74.53 (11.36)	6.40 (1.56)	6.30 (1.97)	6.50 (1.45)	8.20 (1.23)	7.20 (1.86)	7.70 (1.63)	**12.10 (1.56)**	13.90 (2.10)	11.00 (1.91)
No	77	89	74.21 (11.91)	73.23 (12.97)	71.61 (13.53)	4.87 (0.48)	5.42 (0.47)	5.94 (0.44)	6.45 (0.49)	6.48 (0.46)	7.21 (0.51)	**15.59 (0.57)**	12.74 (0.69)	12.34 (0.68)
**Education**														
Lower than secondary education	12	14	66.15 (15.54)	**64.71 (18.72)**	65.63 (15.29)	6.08 (4.64)	**7.92 (5.26)**	7.67 (4.40)	7.58 (5.70)	8.33 (5.07)	8.17 (4.04)	14.67 (6.23)	11.00 (6.94)	11.33 (6.79)
Secondary education	15	17	76.04 (12.88)	**75.10 (12.67)**	73.02 (12.85)	6.33 (5.29)	**6.73 (4.17)**	6.93 (3.97)	7.73 (4.40)	7.13 (4.63)	6.87 (4.29)	14.20 (6.18)	12.27 (6.50)	12.60 (6.43)
Higher than secondary education	60	69	74.66 (10.70)	**75.16 (11.03)**	72.94 (12.83)	4.52 (3.87)	**4.73 (4.07)**	5.43 (3.80)	6.20 (3.90)	6.07 (3.94)	7.18 (4.77)	15.50 (4.55)	13.40 (5.84)	12.25 (5.81)
**Family status**														
Single	33	38	74.01 (12.12)	72.82 (12.09)	69.98 (13.69)	5.82 (4.77)	6.24 (4.67)	6.85 (4.45)	7.79 (4.86)	7.76 (4.80)	8.61 (5.05)	14.36 (5.02)	11.61 (6.11)	10.58 (6.05)
Married or registered partnership	40	46	75.04 (11.32)	74.57 (13.68)	73.87 (12.78)	4.25 (3.56)	4.80 (3.60)	5.48 (3.36)	6.08 (3.62)	6.08 (3.53)	6.80 (3.90)	15.38 (4.95)	13.53 (5.67)	12.65 (5.66)
Divorced, separated or widowed	10	11	67.05 (14.08)	73.01 (14.97)	70.88 (14.48)	6.09 (4.93)	6.27 (5.78)	5.45 (4.20)	6.00 (4.24)	5.36 (4.43)	5.18 (4.71)	15.82 (5.67)	13.55 (6.90)	14.91 (6.07)
Missing	3	3	77.60 (11.73)	74.48 (8.02)	71.88 (14.32)	3.33 (4.16)	4.33 (5.77)	5.67 (5.51)	4.33 (4.51)	4.33 (5.13)	6.33 (4.51)	18.67 (5.51)	15.67 (9.45)	13.67 (7.57)
**Have children under 18 years**														
Yes, under 6 years	5	6	69.38 (5.25)	70.94 (9.48)	73.44 (9.44)	7.00 (5.61)	7.00 (5.70)	6.20 (4.09)	7.80 (4.44)	8.20 (3.56)	7.40 (3.44)	12:00 (4.00)	9.40 (7.33)	10.60 (5.46)
Yes, between 6–14 years	14	16	71.09 (15.20)	75.00 (9.98)	73.44 (13.87)	5.71 (3.99)	5.71 (3.60)	5.79 (4.06)	8.00 (4.87)	6.79 (4.79)	6.86 (5.04)	14:00 (5.39)	12.29 (4.66)	13.71 (4.95)
Yes, between 15–18 years	4	5	75.78 (4.51)	69.53 (11.16)	69.53 (8.32)	3.50 (1.29)	7.75 (2.63)	7.50 (2.89)	6.25 (0.96)	10.00 (2.71)	10.25 (3.20)	16.50 (5.07)	11.25 (4.03)	9.00 (4.08)
No	64	73	74.51 (12.04)	73.90 (13.94)	71.66 (13.81)	4.84 (4.35)	5.22 (4.53)	5.94 (4.05)	6.30 (4.24)	6.17 (4.21)	7.16 (4.62)	15.58 (5.04)	13.38 (6.38)	12.17 (6.32)
Social support* throughout the pandemic														
Moderate to low level	21	28	**67.49 (12.71)**	**68.93 (10.75)**	**62.75 (13.91)**	6.62 (4.50)	**7.12 (4.24)**	**8.20 (4.87)**	6.86 (3.79)	7.54 (4.49)	**8.36 (4.38)**	13.52 (5.37)	10.96 (6.79)	9.56 (5.86)
High level	66	72	**75.71 (11.27)**	**75.74 (13.34)**	**75.66 (11.12)**	4.55 (4.10)	**4.84 (4.30)**	**5.11 (3.16)**	6.59 (4.43)	6.15 (4.12)	**6.82 (4.60)**	15.68 (4.89)	13.69 (5.65)	13.24 (5.76)
**Past mental health treatment**														
Yes	16	18	**66.41 (13.57)**	**63.28 (17.70)**	**58.89 (14.68)**	**7.63 (4.83)**	**7.56 (4.88)**	**9.25 (4.20)**	**9.25 (5.43)**	**9.06 (4.58)**	**9.94 (3.99)**	**11.63 (4.84)**	10.69 (6.70)	**7.19 (4.43)**
No	71	82	**75.37 (11.18)**	**76.06 (10.41)**	**74.89 (11.07)**	**4.46 (394)**	**5.06 (4.16)**	**5.27 (3.54)**	**6.07 (3.76)**	**6.00 (4.00)**	**6.66 (4.49)**	**15.96 (4.80)**	13.37 (5.89)	**13.31 (5.74)**
**Categorised as ‘at risk’ group**														
Yes	18	20	69.10 (14.25)	69.63 (17.31)	69.62 (16.10)	6.72 (5.98)	5.94 (4.14)	6.72 (4.78)	7.94 (5.76)	7.00 (4.69)	7.44 (5.33)	14.39 (5.96)	12.19 (6.51)	11.78 (7.09)
No	69	80	74.93 (11.26)	74.63 (11.71)	72.55 (12.50)	4.61 (3.63)	5.42 (4.46)	5.81 (3.74)	6.32 (3.76)	6.46 (4.18)	7.22 (4.39)	15.36 (4.84)	13.03 (6.04)	12.29 (5.73)
**Categorised as ‘key worker’**														
Yes	39	44	73.00 (13.16)	73.82 (14.80)	**68.67 (15.39)**	5.44 (4.33)	6.19 (5.09)	6.62 (4.52)	7.08 (4.15)	7.32 (4.56)	**8.49 (4.95)**	14.38 (4.95)	12.03 (6.33)	11.03 (6.35)
No	47	54	74.30 (11.37)	73.23 (11.73)	**74.90 (10.63)**	4.74 (4.28)	5.17 (3.81)	5.38 (3.35)	6.30 (4.41)	6.13 (4.01)	**6.13 (3.93)**	15.68 (5.12)	13.39 (5.99)	13.17 (5.63)
Missing	1	2												
**Perception of the level of government interventions**														
Sufficient	35	40	**77.10 (8.74)**	**76.88 (11.60)**	**77.14 (7.82)**	4.03 (3.62)	4.66 (3.89)	**4.40 (2.57)**	6.29 (4.62)	5.86 (4.39)	**5.57 (3.85)**	15.77 (5.02)	**14.68 (5.33)**	13.49 (5.13)
Not sufficient	51	59	**71.54 (13.62)**	**69.82 (13.84)**	**68.17 (15.02)**	5.63 (4.54)	6.47 (4.79)	**7.12 (4.41)**	6.94 (4.06)	7.11 (3.90)	**8.45 (4.72)**	14.82 (5.13)	**10.92 (6.14)**	11.43 (6.41)
Missing	1	1												

Means and SD in parentheses (); statistically significant coefficients (p < 0.05) in bold; p < 0.05, based on t-test or ANOVA; *based on MSPSS score (scores ranging from 1 to 5 were categorised as moderate to low level of support; whilst scores from 5.1 to 7 could be considered high level of support).

*OxCAP-MH* Oxford CAPabilities questionnaire-Mental Health, *HADS-D* Hospital Anxiety and Depression Scale Depression subscale, *HADS-A* Hospital Anxiety and Depression Scale Anxiety subscale, *WHO-5* World Health Organisation-Five Well-being Index.

The results of the univariate analysis, i.e. OxCAP-MH, HADS-D, HADS-A and WHO-5 instrument scores by timepoint and demographic/socioeconomic characteristics showed that significant differences were observed across the three lockdowns. At each consecutive timepoint, the study sample had poorer capability, mental health and wellbeing scores, especially people with past mental health treatment. But reductions were also observed in case of females, people aged 50–64 and those with children aged between 15 and 18 years. Further details can be seen in Table 1.

The random effect models on panel data also found that people with mental health treatment prior to the pandemic experienced significant reduction in their capability, depression, anxiety and general wellbeing scores over time. Satisfaction with government measures was associated with significant improvements in the associations between capability wellbeing, anxiety, and mental wellbeing across lockdowns. High level of social support seems to have influenced the general wellbeing, but not the mental health scales. Depression increased for those with children under 18, whilst women experienced deterioration in their general wellbeing. Further details across the three lockdowns by sociodemographic characteristics can be seen in Table 2.

**Table 2:** Associations between capability wellbeing, depression, anxiety, and general wellbeing across the three lockdowns by sociodemographic characteristics (n = 87).

Sociodemographic characteristics	OxCAP-MH score		HADS-D score		HADS-A score		WHO-5 score	
	ß (SE)	95% CI	ß (SE)	95% CI	ß (SE)	95% CI	ß (SE)	95% CI
Age	− 0.12 (0.10)	− 0.31; 0.07	0.02 (0.03)	− 0.05; 0.09	− 0.02 (0.04)	− 0.09; 0.05	0.01 (0.04)	− 0.08; 0.09
Female	− 0.51 (2.52)	− 5.45; 4.43	0.29 (0.88)	− 1.44; 2.02	0.79 (0.93)	− 1.03; 2.61	− 2.40 (1.09)*	− 4.54; − 0.25
Higher education	0.34 (2.42)	− 4.39; 5.08	− 1.52 (0.84)	− 3.18; 0.13	− 0–47 (0.89)	− 2.22; 1.27	− 0.07 (1.05)	− 2.14; 1.99
Migrant background	0.47 (3.42)	− 6.23 (7.18)	1.55 (1.20)	− 0.80; 3.90	1.63 (1.26)	− 0.85; 4.10	− 2.19 (1.48)	− 5.09; 0.72
Living with partner	2.42 (2.52)	− 2.52; 7.36	− 1.46 (0.88)	− 3.18; 0.26	− 1.27 (0.93)	− 3.09; 0.55	0.60 (1.11)	− 1.57; 2.77
Having a child under 18	− 3.49 (2.72)	− 8.82; 1.84	1.86 (0.95)*	0.00; 3.72	1.96 (1.00)	− 0.01; 3.93	− 1.84 (1.19)	− 4.17; 0.49
High level of social support	4.12 (1.69)*	0.81; 7.42	− 0.89 (0.54)	− 1.96; 0.17	− 0.14 (0.60)	− 1.31; 1.03	1.98 (0.87)*	0.28; 3.68
Past mental health treatment	− 12.39 (2.87)***	− 18.02; − 6.76	2.57 (1.00)*	0.60; 4.54	2.85 (1.06)**	0.78; 4.93	− 3.94 (1.25)**	− 6.39; − 1.49
Risk group	− 1.37 (2.26)	− 5.81; 3.06	0.42 (0.76)	− 1.07; 1.90	0.36 (0.82)	− 1.25; 1.96	− 0.41 (1.06)	− 2.49; 1.67
Key worker	− 0.41 (1.89)	− 4.11; 3.29	0.97 (0.63)	− 0.26; 2.21	1.23 (0.68)	− 0.10; 2.57	− 1.09 (0.89)	− 2.83; 0.64
Knowing someone who died of COVID-19	2.14 (1.73)	− 1.25; 5.53	− 0.32 (0.56)	− 1.42; 0.79	0.13 (0.62)	− 1.08; 1.34	0.69 (0.87)	− 1.02; 2.39
Satisfied with government measures	6.04 (1.55)***	2.99; 9.08	− 1.11 (0.51)	− 2.10; − 0.11	− 1.50 (0.56)**	− 2.59; − 0.40	1.86 (0.77)*	0.35; 3.37
Constant	74.48 (5.31)***	64.06; 84.89	5.80 (1.85)***	2.18; 9.42	7.10 (1.96)***	3.27; 10.94	14.04 (2.35)***	9.44; 18.65

Standard errors (SE) in parentheses * p < 0.05, ** p < 0.01, *** p < 0.001; Statistically significant coefficients (p < 0.05) in bold.

*OxCAP-MH* Oxford CAPabilities questionnaire-Mental Health, *HADS-D* Hospital Anxiety and Depression Scale Depression subscale, *HADS-A* Hospital Anxiety and Depression Scale Anxiety subscale, *WHO-5* World Health Organisation-Five Well-being Index; Level of social support was based on MSPSS score (scores ranging from 1 to 5 were categorised as moderate to low level of support; whilst scores from 5.1 to 7 could be considered high level of support).

The detailed investigation of the differential impacts on the OxCAP-MH capability items found significant reductions in how respondents perceived their social networks. Although not statistically significant, reductions were also observed e.g. in terms of the likelihood of discrimination and assault, influencing local decisions and losing sleep over worries. However, some improvements were reported, for instance, in the appreciation of nature, access to interesting activities or employment, self-determination and feeling safe (Table 3).

**Table 3:** Limitations in capability dimensions based on scores of the OxCAP-MH items at (and between) three waves of data collection (n = 87).

OxCAP-MH items	1st wave*	Change scores (April 2020–November 2020)*	2nd wave*	Change scores (November 2020–December 2021)*	3rd wave*	Change scores (April 2020–December 2021)*
Limitation in daily activities	4.06 (1.13)	0.06	4.11 (1.07)	− 0.13	3.99 (1.07)	− 0.07
Social networks	3.46 (1.34)	**− 0.46**	3.00 (1.35)	**− 0.22**	2.78 (1.05)	**− 0.68**
Losing sleep over worry	3.74 (1.18)	− 0.10	3.63 (1.09)	− 0.06	3.57 (1.19)	− 0.16
Enjoying social and recreational activities	2.74 (1.18)	0.10	2.84 (1.15)	0.00	2.84 (0.96)	0.10
Having suitable accommodation	1.80 (1.07)	− 0.03	1.77 (0.96)	0.05	1.82 (0.87)	0.01
Feeling safe	1.44 (1.44)	− 0.07	1.37 (0.57)	0.20	1.56 (0.90)	0.13
Likelihood of assault	4.45 (0.79)	− 0.17	4.28 (0.90)	− 0.07	4.21 (0.85)	− 0.24
Likelihood of discrimination	4.17 (1.13)	− 0.24	3.93 (1.13)	− 0.06	3.87 (1.27)	− 0.30
Influencing local decisions	3.55 (3.55)	− 0.10	3.45 (1.16)	− 0.08	3.37 (0.99)	− 0.18
Freedom of expression	1.75 (1.00)	0.06	1.80 (1.18)	0.20	2.00 (1.23)	0.25
Appreciating nature	1.28 (0.58)	0.06	1.33 (0.74)	0.00	1.33 (0.66)	0.06
Respecting and valuing people	1.46 (0.71)	− 0.05	1.41 (0.66)	0.14	1.55 (0.68)	0.09
Enjoying friendship and support	2.02 (0.91)	0.00	2.02 (0.98)	0.08	2.10 (0.96)	0.08
Self-determination	2.01 (0.98)	− 0.01	2.00 (1.02)	0.17	2.17 (1.05)	0.16
Imagination and creativity	2.01 (0.95)	− 0.06	1.95 (0.89)	0.16	2.11 (0.96)	0.10
Access to interesting activities or employment	1.71 (0.98)	0.11	1.83 (0.94)	0.13	1.95 (0.90)	0.24

*Higher OxCAP-MH scores mean higher capabilities in that particular item (on a 1–5 likert scale); Statistically significant (p < 0.05) associations in bold.

**Figure 2:**
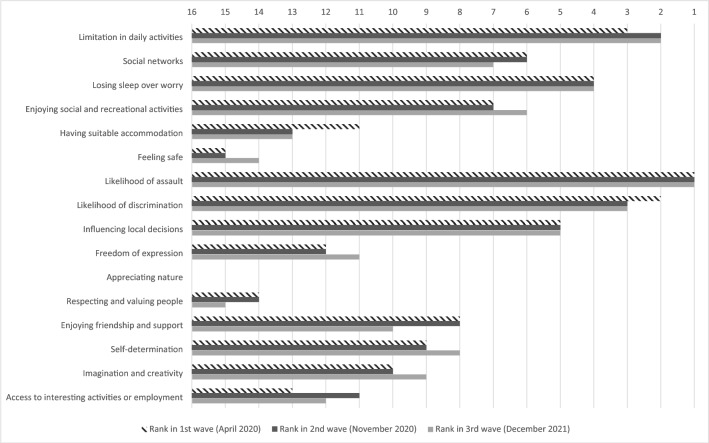
Ranking of OxCAP-MH items at each wave of data collection. OxCAP-MH—Oxford CAPabilities questionnaire-Mental Health; longer bars mean higher capabilities and less limitations.

A visual presentation of the ranks of individual OxCAP-MH items at each timepoint can be seen on Fig. 2. This shows that the highest ranked capability items at each timepoint were the (lack of) limitation in daily activities, likelihood of assault and discrimination. The lowest ranked capability items at each lockdown included feeling safe, appreciating nature and respecting and valuing people.

Associations between change scores of the individual OxCAP-MH capability items and sociodemographic characteristics across the three lockdowns found diverse outcomes across subgroups (Table 4). People with mental health treatment prior to the pandemic reported significantly more limitations in most capability items across the lockdowns than the rest of the cohort. Those with a high level of social support had significantly better outcomes in terms of the likelihood of both discrimination and assault, respecting/valuing people and enjoying friendship/support than those without. Respondents who were satisfied with government measures reported improvements in sleep issues, enjoying social and recreational activities, feeling safe, respecting/valuing people, enjoying friendship/support, self-determination, imagination/creativity and access to interesting activities or employment. The latter one was also felt by people with higher education and those living with a partner. Women’s appreciation of nature and their accommodation increased over time, whilst having a child under 18 reduced the feeling of self-determination. Respondents in an ‘at risk’ group felt that their limitation in daily activities increased. Those defined as key workers lost significantly more sleep, felt less safe and able to decide how they live their lives. Further details can be seen in Table 4.

**Table 4:** Associations between items of the OxCAP-MH instrument across the three lockdowns by sociodemographic characteristics (n = 87).

	Age	Female	Higher education	Migrant background	Living with partner	Having a child under 18	High level of social support	Past MH treatment	Risk group	Key worker	Knowing someone who died of COVID-19	Satisfied with government measures	Constant
Limitation in daily activities								− 0.99 (0.22)	− 1.16 (0.17)				3.91 (0.41)
Social networks													3.03 (0.53)
Losing sleep over worry								− 0.96 (0.27)		− 0.48 (0.18)		0.44 (0.15)	4.57 (0.5)
Enjoying social and recreational activities												0.33 (0.16)	3.05 (0.49)
Having suitable accommodation		0.44 (0.21)											3.34 (0.45)
Feeling safe								− 0.41 (0.16)		− 0.23 (0.12)		0.24 (0.1)	4.44 (0.3)
Likelihood of assault							0.39 (0.14)						4.03 (0.36)
Likelihood of discrimination							0.68 (0.18)						3.47 (0.46)
Influencing local decisions													2.44 (0.47)
Freedom of expression	− 0.02 (0.01)												5.04 (0.53)
Appreciating nature		0.31 (0.13)*						− 0.42 (0.15)					4.71 (0.29)
Respecting and valuing people							0.36 (0.11)	− 0.35 (0.14)				0.21 (0.1)	4.16 (0.27)
Enjoying friendship and support							0.6 (0.14)	− 0.43 (0.2)				0.36 (0.12)	3.69 (0.37)
Self-determination	− 0.02 (0.01)					− 0.66 (0.21)		− 0.9 (0.22)		− 0.32 (0.15)		0.35 (0.13)	4.85 (0.41)
Imagination and creativity								− 0.68 (0.2)				0.34 (0.13)	4.31 (0.38)
Access to interesting activities or employment	− 0.02 (0.01)		0.32 (0.16)		0.33 (0.17)			− 0.57 (0.19)			0.28 (0.14)	0.41 (0.12)	4.41 (0.35)

Standard errors (SE) in parentheses; Only statistically significant (p < 0.05) associations displayed; OxCAP-MH—Oxford CAPabilities questionnaire-Mental Health; Level of social support was based on MSPSS score (scores ranging from 1 to 5 were categorised as moderate to low level of support; whilst scores from 5.1 to 7 could be considered high level of support).

## Discussion

This is the first study to assess the differential capability limiting aspects of lockdowns over time alongside their impacts on mental health and general wellbeing. The results of this study suggest that mental health treatment prior to the pandemic, social support and satisfaction with government measures are the most influential characteristics that determine the association with impacts of the chain of lockdowns. Several previous studies drew attention to mentally ill and socioeconomically deprived groups being the most vulnerable population in terms of impacts of COVID-19^11,14,41–43^. Mental health patients were found to be vulnerable during the pandemic because of several reasons, including somatic vulnerability, cognitive and behavioural vulnerability, psychosocial vulnerability, and disruption to psychiatric care^9^. Previous studies assessing the impacts of COVID-19 found that young people, rather than older people, are most vulnerable to increased psychological distress, and especially young women and people with young children are at particularly high risk for mental-health problems due to the COVID-19 pandemic^14,42^. Despite the small sample, our study found that respondents with children aged between 15 and 18 years showed constant deterioration. This could be explained by the enormous number of home schooling days experienced by this group^44^. Our study confirmed the findings of other Austrian studies which found that the experience of loneliness was associated with poorer emotional wellbeing during the pandemic^45,46^. This could also explain why people without children under 18 years of age showed constantly deteriorating capability, mental health and wellbeing outcomes. In addition, previous studies investigated perceived stress status and post-traumatic stress disorder symptoms among individuals quarantined/isolated during the COVID-19 pandemic, and they identified correlations with religious practices or whether individuals were in forced or voluntary quarantine/isolation^48,49^. These aspects of wellbeing are also linked to freedom of choice, highlighting the importance of the capabilities approach in understanding the impacts of lockdowns.

When exploring the deeper impacts on individual capabilities, our results are in line with the findings of the Austrian Corona Panel Project, which found that the extended lockdowns lead to a decreased level of solidarity and tolerance towards other people in the society compared to the beginning of the pandemic^47,48^. The mixed impacts of high social support on how people experienced the lockdowns could be linked to the known associations with close social contacts. There was significant deterioration in terms of social networks, paired with a perceived increased likelihood of assault and the ability to influence local decisions. Although social support helped most people in their everyday life during the pandemic, they also felt that their flat situation became unsuitable and they had less options to express themselves or be creative. Moreover, people around them and their love and support became a burden. In extreme cases, this could even be linked to the increase in domestic violence cases that were amplified during the recent pandemic because the victim remained trapped inside the home with the abuser, with no escape route or the opportunity to contact outside help^49,50^. This could also explain why our unadjusted analysis found increasing capability, mental health and wellbeing burden for women.

Improvements in some capability items could be observed for people with certain attributes. A significant reduction was captured in social networks, but a notable drop in the perceived likelihood of assault/discrimination, and the ability to influence local decisions was also observed. The OxCAP-MH items also detected that respondents’ perception of the freedom of expression, self-determination and access to interesting activities and employment improved over the subsequent lockdowns. Respondents with high social support reported that it was less likely that they experience assault or discrimination and experienced increase in capability aspects such as respecting/valuing people and enjoying friendship/support. Respondents who were satisfied with the interventions of the government also reported better scores in most capability aspects compared to those who were rather critical. These findings highlight the heterogenous experiences people gathered during the lockdowns, and the multifaceted aspects that should be considered when assessing the impacts of the COVID-19 crisis. It is well known that people adapt quickly and inevitably to any life changes^51^. This could also explain the findings of a longitudinal Austrian study which also used WHO-5 and found that although the detrimental health consequences of the COVID-19 pandemic persisted for several months, there was a slight trend toward improvement in wellbeing scores^52^. In addition, opportunities for vaccination appeared between the second and third wave of data collection which could have caused some relief for some respondents and extra burden for others. Government measures also became less restrictive in certain areas of life. For instance, travelling became easier for those who were tested and later vaccinated. This could explain the initial shock for people with migrant background found in the unadjusted analysis which later resolved.

The quantification of mental health and wellbeing consequences of the pandemic mitigation measures is known to be challenging because it requires a longitudinal analysis that can track the relatively rapid changes that mitigation policies could produce and the duration of the changes^53^. The main strength of our study is that it followed up a group of people in a longitudinal analysis across three lockdowns comparable in length and strength, while the overall pandemic impacts had major developments. Furthermore, although our study is only exploratory in nature due to the limited sample size, we conducted robust statistical analysis methods and explored the impacts of the lockdowns in a comprehensive way including also a unique capabilities approach angle. The main limitations of this study is the limited sample which calls for caution when interpreting results for sub-groups that had very small sample size such as the youngest and oldest age cohorts. In addition, the 95% CI was sometimes very wide which indicates that the conclusions are less certain, however, this is in line with the exploratory nature of the study. Further limitations can be attributed to online data collection which is prone to self-reporting bias^54^; and the over-representation of certain sociodemographic groups^55,56^. Nevertheless, we approached diverse population groups in a targeted manner, for instance, through advertising the survey in the comment sections of local newspaper articles on Facebook. As a result, the survey achieved satisfactory representation in terms of more than half of the Austrian provinces, migration background and employment status^8^. A further limitation is that the recruitment strategy and the topic generated an overrepresentation of past mental health users in the sample, compared to the general population, and these participants were also more motivated to participate in the second round of data collection. Nevertheless, this could also be perceived as strength of this study because despite the small sample size, we have achieved a good coverage of those who were previously impacted by mental health issues.

## Implications and conclusions

The assessment of the capability limiting aspects of subsequent lockdowns alongside their impacts on mental health and general wellbeing found that in order to increase public resilience and compliance with interventions, future public health policies concerning lockdowns should pay special attention to people with mental health treatment prior to the pandemic, those characterised by relatively low level of social support and by lack of satisfaction with government measures.

## Data Availability

The datasets generated during the current study and the study protocol is available on request.

## Abbreviations

HADS: The Hospital Anxiety and Depression Scale
MSPSS: The Multidimensional Scale of Perceived Social Support
OxCAP-MH: The Oxford CAPabilities questionnaire-Mental Health
WHO-5: The World Health Organisation-Five Wellbeing Index

## References

[1] UNDP. COVID-19 and human development: Assessing the crisis, envisioning the recovery. in *2020 Human Development Perspectives.* Edited by Programme UND; 2020.

[2] Agha S (2021). Mental well-being and association of the four factors coping structure model: A perspective of people living in lockdown during COVID-19. Ethics Med. Public Health.

[3] Nations U. UN leads call to protect most vulnerable from mental health crisis during and after COVID-19. *UN News* 2020.

[4] Jeong H, Yim HW, Song YJ, Ki M, Min JA, Cho J, Chae JH (2016). Mental health status of people isolated due to Middle East Respiratory Syndrome. Epidemiol. Health.

[5] Hossain, M., Patwary, M.M., Sultana, R., Browning, M.H. Psychological distress among healthcare professionals during the early stages of the COVID-19 outbreak in low resource settings: A cross-sectional study in Bangladesh. *Front. Public Health*. 1742 (2021). 10.3389/fpubh.2021.701920 PMC8632035 34858914

[6] Patwary, M.M., Bardhan, M., Disha, A.S., Kabir, M.P., Hossain, M.R., Alam, M.A., Haque, M.Z., Billah, S.M., Browning, M., Kabir, R. *et al*. Mental health status of university students and working professionals during the early stage of COVID-19 in Bangladesh. *Int. J. Environ. Res. Public Health*. **19**(11) (2022). 10.3390/ijerph19116834 PMC9180371 35682415

[7] Jain A, Bodicherla KP, Raza Q, Sahu KK (2020). Impact on mental health by “Living in Isolation and Quarantine” during COVID-19 pandemic. J. Family Med. Primary Care.

[8] Cavicchioli M, Ferrucci R, Guidetti M, Canevini MP, Pravettoni G, Galli F (2021). What will be the impact of the COVID-19 quarantine on psychological distress? Considerations based on a systematic review of pandemic outbreaks. Healthcare (Basel).

[9] Sheek-Hussein M, Abu-Zidan FM, Stip E (2021). Disaster management of the psychological impact of the COVID-19 pandemic. Int. J. Emerg. Med..

[10] Boden M, Zimmerman L, Azevedo KJ, Ruzek JI, Gala S, Abdel Magid HS, Cohen N, Walser R, Mahtani ND, Hoggatt KJ (2021). Addressing the mental health impact of COVID-19 through population health. Clin. Psychol. Rev..

[11] Simon J, Helter TM, White RG, van der Boor C, Łaszewska A (2021). Impacts of the COVID-19 lockdown and relevant vulnerabilities on capability well-being, mental health and social support: An Austrian survey study. BMC Public Health.

[12] Kola L, Kola BA, Hanlon C, Naslund JA, Sikander S, Balaji M, Benjet C, Cheung EYL, Eaton J, Gonsalves P, Hailemariam M, Luitel NP, Machado DB, Misganaw E, Omigbodun O, Roberts T, Salisbury TT, Shidhaye R, Sunkel C, Ugo V, Van Rensburg AJ, Gureje O, Pathare S, Saxena S, Thornicroft G, Patel V (2021). COVID-19 mental health impact and responses in low-income and middle-income countries: reimagining global mental health. Lancet Psychiatry..

[13] Reid, J.M.K., Siepmann, I., Mason-Jones, A., Simon, J. (ASPHER COVID-19 Task Force): Building forwards fairly 2022–2032: What can we learn from COVID-19’s unequal and unjust losses in Europe? in *Second ASPHER Statement on the Pandemic Impacts on Health Inequalities in Disadvantaged Vulnerable Populations in the European Region*. (2022).

[14] Force AC-T. What are the COVID-19 Lockdown-induced illnesses and why should European public health systems be investigating their epidemiology, treatment, and prevention? (2020).

[15] Prati G, Mancini AD (2021). The psychological impact of COVID-19 pandemic lockdowns: A review and meta-analysis of longitudinal studies and natural experiments. Psychol. Med..

[16] Jolly, T.S., Pandian, G., Batchelder, E., Jain, A. Posttraumatic stress disorder exacerbation as a result of public masking in times of COVID-19. *Prim. Care Companion CNS Disord*. **22**(6) (2020). 10.4088/PCC.20l02828 33369294

[17] Laboe CW, Jain A, Bodicherla KP, Pathak M (2021). Physician suicide in the era of the COVID-19 pandemic. Cureus.

[18] Sabat I, Neuman-Böhme S, Varghese NE, Barros PP, Brouwer W, van Exel J, Schreyögg J, Stargardt T (2020). United but divided: Policy responses and people’s perceptions in the EU during the COVID-19 outbreak. Health Policy.

[19] Moshammer, H., Poteser, M., Lemmerer, K., Wallner, P., Hutter, H.P. Time course of COVID-19 cases in Austria. *Int. J. Environ. Res. Public Health*. **17**(9) (2020). 10.3390/ijerph17093270 PMC7246438 32392880

[20] Nagel, A., Łaszewska, A., Haidinger, G. & Simon, J. The first 8 weeks of the Austrian SARS-CoV-2 epidemic. *Wien Klin Wochenschr.* **133**(7-8), 364–376. 10.1007/s00508-020-01804-9 (2021). 10.1007/s00508-020-01804-9 PMC7848873 33523297

[21] Łaszewska A, Helter T, Simon J (2021). Perceptions of COVID-19 lockdowns and related public health measures in Austria: A longitudinal online survey. BMC Public Health.

[22] Yuan, K., Gong, Y.M., Liu, L., Sun, Y.K., Tian, S.S., Wang, Y.J., Zhong, Y., Zhang, A.Y., Su, S.Z., Liu, X.X. *et al*. Prevalence of posttraumatic stress disorder after infectious disease pandemics in the twenty-first century, including COVID-19: A meta-analysis and systematic review. *Mol. Psychiatry*. (2021). 10.1038/s41380-021-01036-x PMC7861006 33542468

[23] Richter D, Riedel-Heller S, Zürcher SJ (2021). Mental health problems in the general population during and after the first lockdown phase due to the SARS-Cov-2 pandemic: Rapid review of multi-wave studies. Epidemiol. Psychiatric Sci..

[24] Liu, S., Heinz, A., Haucke, M.N., Heinzel, S. The global impact of the COVID-19 pandemic on the care provision for people with mental health problems. *Nervenarzt*. 1–5 (2021). 10.1007/s00115-021-01068-2 PMC7877534 33575836

[25] White, R.G., Imperiale, M.G., Perera, E. The capabilities approach: Fostering contexts for enhancing mental health and wellbeing across the globe. *Global. Health*. **12**(1) (2016). 10.1186/s12992-016-0150-3 PMC4858826 27150600

[26] Sen, A. Commodities and capabilities. (North-Holland Sole distributors for the U.S.A. and Canada, Elsevier Science Pub. Co., 1985).

[27] Sen A (1999). Development as Freedom.

[28] Probst T, Budimir S, Pieh C (2020). Depression in and after COVID-19 lockdown in Austria and the role of stress and loneliness in lockdown: A longitudinal study. J. Affect. Disord..

[29] Middleton J, Lopes H, Michelson K, Reid J (2020). Planning for a second wave pandemic of COVID-19 and planning for winter. Int. J. Public Health.

[30] Simon JAP, Gray A, Rugkasa J, Yeeles K, Burns T (2013). Operationalising the capability approach for outcome measurement in mental health research. Social Sci. Med..

[31] Simon, J., Łaszewska, A., Leutner, E., Spiel, G., Churchman, D., Mayer, S. Cultural and linguistic transferability of the multi-dimensional OxCAP-MH capability instrument for outcome measurement in mental health: The German language version. *BMC Psychiatry*. **18**(1) (2018). 10.1186/s12888-018-1762-3 PMC5987381 29866092

[32] Vergunst, F., Jenkinson, C., Burns, T., Anand, P., Gray, A., Rugkåsa, J., Simon, J. Psychometric validation of a multi-dimensional capability instrument for outcome measurement in mental health research (OxCAP-MH). *Health Qual. Life Outcomes*. **15**(1) (2017). 10.1186/s12955-017-0825-3 PMC5745777 29282075

[33] Łaszewska A, Schwab M, Leutner E, Oberrauter M, Spiel G, Simon J (2019). Measuring broader wellbeing in mental health services: Validity of the German language OxCAP-MH capability instrument. Qual. Life Res..

[34] Snaith RP, Zigmond AS (1986). The hospital anxiety and depression scale. Br. Med. J. (Clin. Res. Ed.).

[35] Bjelland I, Dahl AA, Haug TT, Neckelmann D (2002). The validity of the Hospital Anxiety and Depression Scale. An updated literature review. J. Psychosom. Res..

[36] WHO. Wellbeing measures in primary health care/The Depcare Project. Report on a WHO Meeting. Copenhagen (1998).

[37] Topp CW, Østergaard SD, Søndergaard S, Bech P (2015). The WHO-5 Well-Being Index: A systematic review of the literature. Psychother. Psychosom..

[38] Garland AF, Deyessa N, Desta M, Alem A, Zerihun T, Hall KG, Goren N, Fish I (2018). Use of the WHO’s Perceived Well-Being Index (WHO-5) as an efficient and potentially valid screen for depression in a low income country. Fam. Syst. Health.

[39] Zimet GD, Dahlem NW, Zimet SG, Farley GK (1988). The multidimensional scale of perceived social support. J. Pers. Assess..

[40] StataCorp. (2019). Stata Statistical Software: Release 16.

[41] Tsamakis K, Tsiptsios D, Ouranidis A, Mueller C, Schizas D, Terniotis C, Nikolakakis N, Tyros G, Kympouropoulos S, Lazaris A (2021). COVID-19 and its consequences on mental health (Review). Exp. Ther. Med..

[42] Abbott A. COVID's mental-health toll: how scientists are tracking a surge in depression. *Nature.* **590**(7845), 194–195. 10.1038/d41586-021-00175-z (2021). 10.1038/d41586-021-00175-z 33536600

[43] Wu T, Jia X, Shi H, Niu J, Yin X, Xie J, Wang X (2021). Prevalence of mental health problems during the COVID-19 pandemic: A systematic review and meta-analysis. J. Affect. Disord..

[44] Pieh C, Dale R, Plener PL, Humer E, Probst T. Stress levels in high-school students after a semester of home-schooling. *Eur Child Adolesc Psychiatry.* **16**, 1–3. 10.1007/s00787-021-01826-2 (2021). 10.1007/s00787-021-01826-2 PMC8206876 34132922

[45] Stieger S, Lewetz D, Swami V. Emotional Well-Being Under Conditions of Lockdown: An Experience Sampling Study in Austria During the COVID-19 Pandemic. *J Happiness Stud.* **22**(6), 2703–2720. 10.1007/s10902-020-00337-2 (2021). 10.1007/s10902-020-00337-2 PMC7778412 33424431

[46] Stolz E, Mayerl H, Freidl W. The impact of COVID-19 restriction measures on loneliness among older adults in Austria. *Eur J Public Health. ***31**(1), 44–49. 10.1093/eurpub/ckaa238 (2021). 10.1093/eurpub/ckaa238 PMC7799060 33338225

[47] Kittel, B. Die Entsolidarisierung der Gesellschaft: Vom ersten in den zweiten Lockdown. (2020).

[48] Kittel, B., Kritzinger, S., Boomgaarden, H., Prainsack, B., Eberl, J.-M., Kalleitner, F., Lebernegg, N.S., Partheymüller, J., Plescia, C., Schiestl, D.W. *et al*. The Austrian Corona Panel Project: Monitoring individual and societal dynamics amidst the COVID-19 crisis. Eur Polit Sci 20, 318–344. 10.1057/s41304-020-00294-7 (2021).

[49] Bradbury-Jones C, Isham L (2020). The pandemic paradox: The consequences of COVID-19 on domestic violence. J. Clin. Nurs..

[50] Sacco MA, Caputo F, Ricci P, Sicilia F, De Aloe L, Bonetta CF, Cordasco F, Scalise C, Cacciatore G, Zibetti A (2020). The impact of the COVID-19 pandemic on domestic violence: The dark side of home isolation during quarantine. Med. Leg. J..

[51] Brickman, P., Campbell, D.T., Appley, M.H. Adaptation level theory: A symposium. (1971).

[52] Pieh, C., Budimir, S., Humer, E., Probst, T. Comparing mental health during COVID-19 lockdown and six months later in Austria: A longitudinal study. (2020). 10.3389/fpsyt.2021.625973 PMC8042148 33859579

[53] Fayaz Farkhad B, Albarracín D (2021). Insights on the implications of COVID-19 mitigation measures for mental health. Econ. Hum. Biol..

[54] Devaux M, Sassi F (2016). Social disparities in hazardous alcohol use: Self-report bias may lead to incorrect estimates. Eur. J. Public Health.

[55] Mulder, J., de Bruijne, M. Willingness of online respondents to participate in alternative modes of data collection. *Survey Practice*. **12**(1) (2019).

[56] Yetter G, Capaccioli K (2010). Differences in responses to Web and paper surveys among school professionals. Behav. Res. Methods.

